# Revisiting molecular hydrogen signaling in mitochondria: Is the Rieske protein the entry point or a downstream sentinel?

**DOI:** 10.1016/j.redox.2026.104003

**Published:** 2026-01-05

**Authors:** Sergej M. Ostojic

**Affiliations:** Faculty of Health Sciences, University of Pécs, Pécs, Hungary; Applied Bioenergetics Lab, Center for Mitochondrial Medicine, Medical Polyclinic Fizikus, Belgrade, Serbia

**Keywords:** Molecular hydrogen, Rieske iron-sulfur protein, Mitochondrial redox signaling, Electron transport chain, LONP1-Mediated proteostasis, Bioenergetics

## Abstract

A recent study published in *Redox Biology* (Volume 88, December 2025, 103952) demonstrates that molecular hydrogen (H_2_) rapidly suppresses mitochondrial Complex III activity through a mechanism involving the Rieske iron-sulfur protein (RISP) and subsequent LONP1-dependent proteolysis, challenging the long-standing view of H_2_ as merely a selective radical scavenger. While these findings compellingly identify RISP as a key mediator of mitochondrial responses to H_2_, its designation as the primary molecular target warrants broader consideration. From an evolutionary and structural standpoint, RISP belongs to a wider family of hydrogenase-like mitochondrial redox proteins that retain ancient iron-sulfur architectures. Proteins such as succinate dehydrogenase subunit B (SDHB), iron-sulfur subunits of Complex I, and CISD family [2Fe–2S] proteins share comparable redox logic and strategic positioning within mitochondrial bioenergetic networks. Here, these candidates are prioritized and placed into a hierarchical, testable framework, and specific comparative structural, biochemical, and proteostatic approaches are proposed to define the true molecular entry point of H_2_ signaling in human mitochondria.

I read with great interest the recent article reporting that molecular hydrogen (H_2_) rapidly suppresses mitochondrial Complex III activity through a mechanism involving the Rieske iron–sulfur protein (RISP/UQCRFS1) and subsequent LONP1-dependent proteolysis [1]. The authors provide elegant kinetic evidence demonstrating that H_2_ exposure induces a near-immediate reduction in Complex III activity, followed by a pronounced decrease in RISP abundance within 1 h, an effect not reproduced by classical antioxidants such as *N*-acetylcysteine. These findings represent a major advance in hydrogen biology by moving the field beyond the long-standing paradigm of nonspecific radical scavenging and, for the first time, proposing a defined mitochondrial protein involved in H_2_ signaling.

This conceptual shift warrants explicit emphasis. Historically, the biological effects of H_2_ have been attributed largely to selective hydroxyl radical scavenging or indirect modulation of redox-sensitive signaling pathways [2,3], without identification of a discrete molecular entry point. In this context, the demonstration that a specific mitochondrial protein is rapidly and selectively affected by H_2_ provides a long-sought mechanistic anchor for the field. At the same time, the identity of the *primary* mitochondrial target of H_2_ may be context dependent, influenced by cell type, oxygen tension, mitochondrial metabolic state, and the prevailing redox poise of the ubiquinone pool.

From an evolutionary and structural standpoint, RISP is not unique in retaining iron-sulfur (Fe–S) motifs derived from ancient anaerobic oxidoreductases [4]. Several mitochondrial proteins share comparable redox logic, Fe–S cluster density, and strategic positioning within the electron transport chain, making them plausible alternative or co-primary sites of H_2_ action. However, these candidates are not equivalent in their likelihood of acting as the initial molecular entry point.

Among hydrogenase-like proteins, succinate dehydrogenase subunit B (SDHB) emerges as a particularly high-priority comparator. SDHB forms a compact Fe–S relay that directly couples tricarboxylic acid cycle flux to the ubiquinone pool [5], positioning it upstream of Complex III redox control. Even modest H_2_-induced perturbations in SDHB redox tuning could rapidly alter the ubiquinone redox state and secondarily suppress Complex III activity, reproducing the observed phenotype without requiring direct interaction between H_2_ and RISP. Notably, Complex II activity was not directly assessed in the original study, leaving this possibility unresolved.

By contrast, the Fe–S core of Complex I, particularly subunits such as NDUFS1, NDUFS7, and NDUFS8, represents a more distributed and buffered redox system. Although Complex I is widely regarded as an evolutionary remnant of ancestral hydrogenases and retains an extensive low-potential Fe–S cluster chain [6], cluster-specific perturbations may not immediately translate into detectable changes in bulk NADH dehydrogenase activity. Nonetheless, subtle modulation of Complex I Fe–S centers could reshape the redox environment of the ubiquinone pool and influence downstream complexes.

Beyond the canonical respiratory chain, CISD family proteins, including mitoNEET (CISD1) and NAF-1 (CISD2), represent a distinct class of redox-responsive [2Fe–2S] regulators located at mitochondrial membranes [7]. These proteins do not primarily function as electron-transfer conduits but rather as sensors and modulators of mitochondrial redox homeostasis, stress signaling, and proteostasis. In this framework, CISD proteins may act as upstream redox sentinels for H_2_ exposure, initiating signaling cascades that culminate in selective degradation of RISP via LONP1. Thus, RISP loss may represent a prominent downstream execution point rather than the initiating molecular lesion.

These relationships are summarized in Fig. 1, which contrasts the experimentally demonstrated H_2_-RISP-LONP1 axis with a broader network-level model in which multiple hydrogenase-like Fe–S proteins modulate ubiquinone redox state and mitochondrial proteostasis.

**Fig. 1:**
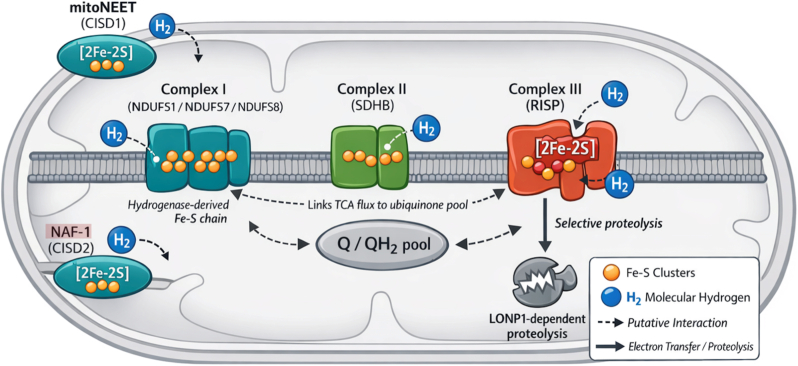
**Putative mitochondrial targets of molecular hydrogen within a hydrogenase-like redox network.** Schematic overview of the mitochondrial electron transport chain highlighting iron-sulfur (Fe–S) proteins with evolutionary or functional similarity to ancestral hydrogenases. Complex I (NDUFS1/7/8), Complex II (SDHB), and Complex III (Rieske iron-sulfur protein; RISP) are shown in relation to the ubiquinone (Q/QH_2_) pool. CISD family proteins (mitoNEET/CISD1 and NAF-1/CISD2) are depicted at the mitochondrial membranes as redox-responsive Fe–S regulators. Putative sites of molecular hydrogen (H_2_) interaction are indicated by dashed arrows. Downstream LONP1-dependent proteolysis of RISP is shown as a potential convergent outcome of upstream H_2_-induced redox perturbations. The figure illustrates how RISP may function either as a primary molecular target of H_2_ or as a downstream sentinel within a broader mitochondrial redox network.

The authors appropriately acknowledge the absence of direct structural or biophysical evidence demonstrating interaction between H_2_ and RISP. Addressing this gap will be essential for establishing molecular primacy. Comparative approaches should prioritize temporal resolution and protein specificity. Parallel assessment of early redox-sensitive modifications, such as Fe–S cluster oxidation state, conformational stability, or local unfolding, across SDHB, RISP, and selected Complex I subunits following acute H_2_ exposure would help identify the earliest responding node. In addition, comparative proteostatic profiling under conditions of LONP1 inhibition or knockdown could clarify whether RISP degradation is unique or part of a broader quality-control response. Finally, reconstitution of individual Fe–S proteins in isolated membrane or proteoliposome systems may allow direct interrogation of H_2_-induced structural or electronic effects independent of network-level buffering.

Importantly, H_2_ signaling does not occur in isolation from other regulatory inputs that shape mitochondrial redox tone. Evolutionarily conserved modulators such as nitric oxide, hydrogen sulfide, calcium, and reactive lipid species influence electron flow, supercomplex assembly, and mitochondrial proteostasis [8]. Crosstalk between these signaling molecules and H_2_ exposure—particularly under intermittent or pulsatile dosing regimens—may critically determine whether H_2_ functions predominantly as a signaling modulator, a hormetic stressor, or a trigger of selective protein turnover.

Finally, the context dependence of RISP targeting warrants explicit consideration. Oxygen tension alters the redox stability of the Rieske [2Fe–2S] cluster, mitochondrial metabolic state determines ubiquinone reduction pressure, and cell type dictates reliance on specific respiratory nodes. Under conditions of high electron flux and a highly reduced ubiquinone pool, RISP may become particularly susceptible to redox-induced destabilization and LONP1-mediated proteolysis. Conversely, in low-flux or hypoxic states, upstream modulation at SDHB or regulatory CISD proteins may predominate. Incorporating these variables into experimental design will be essential for defining when RISP functions as a primary molecular target versus a downstream sentinel of mitochondrial stress.

In summary, the study by Negishi and co-workers [1] compellingly elevates RISP as a central mediator of mitochondrial responses to molecular hydrogen and marks a turning point in hydrogen biology. At the same time, a hierarchical and context-sensitive view of hydrogenase-like mitochondrial redox proteins may better capture the complexity of H_2_ signaling. Systematic, comparative interrogation of SDHB, RISP, Complex I Fe–S subunits, and CISD proteins across defined metabolic and redox states now represents a logical next step toward identifying the true molecular entry point of H_2_ signaling in human mitochondria.

## Statement of ethics

Not applicable.

## Consent for publication

The author gives permission for the Journal to publish this work.

## Funding sources

None.

## Declaration of competing interest

The authors declare that they have no known competing financial interests or personal relationships that could have appeared to influence the work reported in this paper.

## Data Availability

No data was used for the research described in the article.
